# Cardiocentrism in ancient medicines

**DOI:** 10.1016/j.ijcha.2023.101261

**Published:** 2023-08-25

**Authors:** Fabio Zampieri, Gaetano Thiene, Alberto Zanatta

**Affiliations:** Department of Cardiac, Thoracic, Vascular Sciences and Public Health, University of Padua, Italy

**Keywords:** History of medicine, History of cardiology, Anatomy of the heart, Mesopotamian medicine, Egyptian Medicine, Ayurveda, Traditional Chinese medicine

## Abstract

History of cardiology starts scientifically in 1628, when William Harvey (1578–1657) published his revolutionary book *Extercitatio anatomica de motu cordis et sanguinis in animalibus*, where he described “general” circulation, movements and functions of heart, heart valves, veins and arteries [1]. Consequently, all theories and practices of ancient medicines were reduced to superstitions. Historians relegated pre-Harveian cardiology to roughs notes, preventing a proper historical evaluation of many centuries of conceptions and practices. All the ancient civilizations shared the conviction that the heart was the biological and spiritual center of the body, the seat of emotions, mind, will, a vital energy produced by breathing and healing, and the soul. This cardiocentric view maintained a special role both in religion and in medicine across millennia from east to west, passing over cultural and scientific revolutions. Here, we will try to give a schematic account of medical beliefs on the heart from the most important pre-classic medicines. Some of them today show to have a kernel of truth. This demonstrates, at least, that history is a non-linear process and that intuitions or even truths, potentially useful for the present and scientific development, can re-emerge from the past.

## 1 Heart in mesopotamia

It is possible to state that blood and liver had a prominent role in Mesopotamian culture and religion (3000––539 BCE). The blood was the fundamental vital principle of life with a divine nature. In the Old Babylonian *Atrahasis* epic, the gods created humans by mixing clay with the blood of a rebellious deity named *We-ilu*, who was specially slaughtered for the occasion [2].

**Fig. 1:**
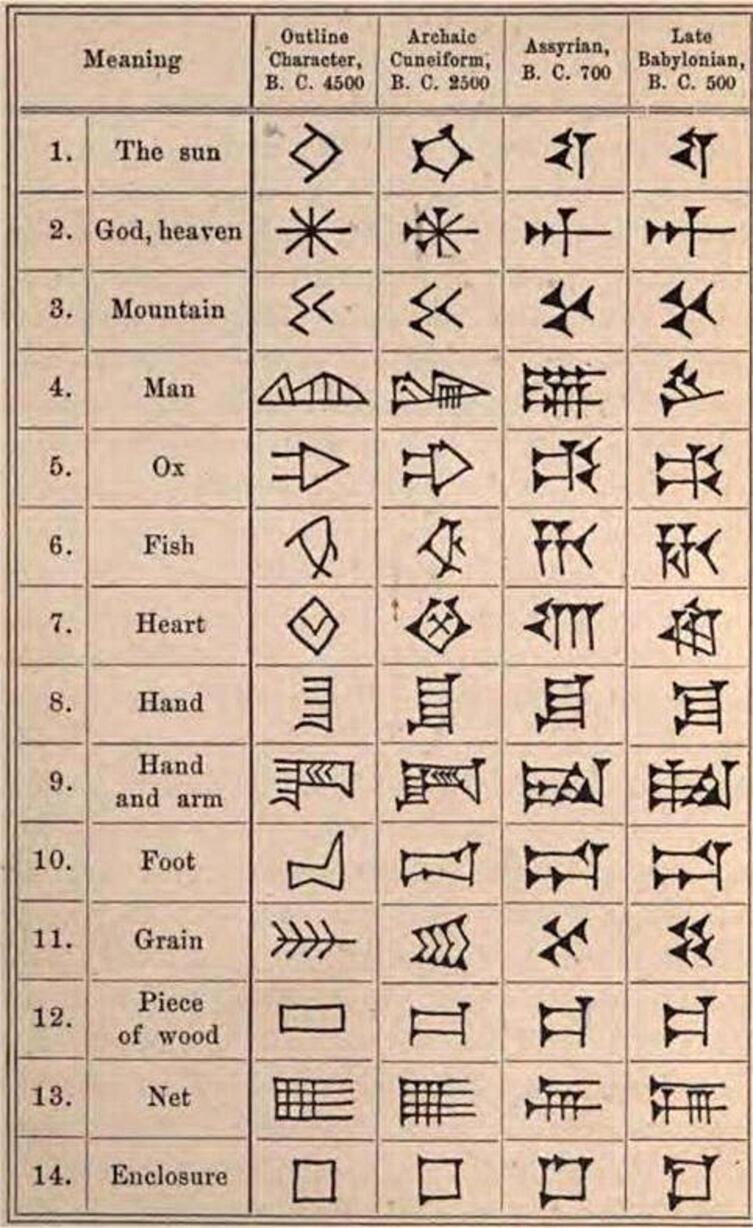
Chronological evolution of the cuneiform characters for the word heart (number 7).

The liver was considered the main organ related to blood. Accordingly, when an animal was sacrificed, its liver was thoroughly examined for finding signs of destiny imposed by divinities. Mesopotamians learned a lot about internal anatomy from sheep. In cuneiform sources, there are many more detailed descriptions of the sheep’s heart than of the human heart. The Akkadian word for heart, *libbu*, refers also to the stomach (Fig. 1). For the heart sheep, we have words for the “rear”, the “upper”, the “middle”, the “apex” and the “thick part of the heart”; the “joint of the heart”; and the “right side of the heart”. The pericardium is called the “fortress of the heart”. Extispicy priests had also terms for the portal vein and vena cava, while there is not even a special word for “vein” or “artery” in human anatomy [3]. The Akkadian term *seranu* might mean “blood vessels” and “sinews”, really any stringy part of the anatomy [4].

Even if the liver seems to have a predominant role, also the heart was important. The term *libbu* could indicate also the abdomen, the viscera, or the uterus [5]. It could mean the “interior” of something, such as a building, area, or region. Conjugated with *bisitu* – meaning “property” – it could indicate an “internal disease” [6]. With the meaning of “interior”, *libbu* was interchangeable with *qirbu*, indicating also “internal organs” [7].

The term *libbu* was also related to mental and emotional states. It could mean mind, thought, intention, courage, will, desire, choice, and preference [6]. Indeed, the *libbu* was the seat of mind or intelligence. This hypothesis seams corroborated by the fact that many Mesopotamian texts describe mental symptoms using *libbu* or *temu*, letting understand that are probably located in the same area [8]. *Temu* was the intelligence or reason of men with a divine origin [9].

Many emotional states were described conjugating *libbu* with other words related to specific emotions. Emotional states were connected also to the liver. The terms *kabittu* (liver) and *libbu* were often used interchangeably in the description of emotions [7]. Kidneys (*kalitu* at singular and *kalate* at plural) are also employed to describe emotions. For instance, the expressions *lipassih kalit-ka*, *lipassih libbi-ka* and *lipassih habitti-ka*, meaning, respectively, “what calms the kidneys”, “what calms the heart” and “what calms the liver” were used for designating a state of calm [7].

The fact that also liver and kidneys were connected to emotions might indicate that, when Mesopotamians used the term *libbu* in relationship to an emotional state, they intended exactly the heart, and not generally the “interior” of the body. At the same time, the fact that only the heart, and not the liver or other organs, was designated with a term characterized by a great richness of meanings indicates that this organ had a special place in the economy of the body. That is, only the heart was indicated with a term equivalent to the interior of the body. This connection might have favoured, in the course of the following cultural history of near civilisations, such as the Egyptian, the Indian and the Chinese ones, the development of the idea that the heart was the spiritual centre of the body, as if the heart became the “interior of the interior of the body”.

Mesopotamian doctors were aware that the heart has a rhythmic movement and that the body has at its interior vessels containing blood. Two diagnostic terms, *esu* and *dalahu*, indicated an irregular rhythm of pathologic nature [10]. In the so-called *Babylonian Diagnostic Handbook,* a “palpitation” of the heart is mentioned in relationship with chest pain, probably in case of myocardial infarction. Interestingly, excess of food and strong emotions were recognized as possible trigger of this condition [10]. Formula speaking of shortness of breath, pain or swelling of viscera, and/or muscular rigidity probably were connected with heart failure [10].

Ghosts could cause heart fluttering and shortness of breath. A prescription mentions juniper and cypress that could have a cardiovascular effect [11]. The use of juniper is approved only for dyspeptic disorders, thanks to the carminative and stomachic effects of the essential oil, as well as to the resinous substances contained in the fruits of the plant. To the juniper are attributed other properties. The essential oil demonstrates to have a diuretic and anti-inflammatory activity realized through the inhibition of the enzyme cyclooxygenase. The main responsible of diuretic effect seems to be the Terpinen-4-ol. Moreover, juniper demonstrates to have hypoglycemic, hypotensive, antiseptic properties, and an antiviral function against the Herpes simplex virus. More clinical studies, however, are needed before approving these therapeutic applications [12]. Cypress seems to favour microcirculation. It seems useful in case of haemorrhoids and peripheral venous insufficiency. Moreover, cypress has an anti-inflammatory activity in the respiratory tract and it probably has also a diuretic function [12].

To sum up, even if the liver might have had a prominent role in Mesopotamian medicine and religion, the analysis of the term *libbu*, of diagnostic and therapeutic texts specifically dedicated to the heart, demonstrates that also that organ had an important role. This model might have inspired, or have been connected, with the cardiocentric model developed in other ancient medicines and cultures, such as the Egyptian one.

## 2 Heart in Egypt

From the Pharaonic Egypt (3000–––332 BCE), we dispose of four manuscripts dedicated to the heart and cardiovascular diseases, contained in the Smith papyrus [13], in the Ebers papyrus [14], [15], and in the papyrus Berlin 3038 [16].

The heart was, at the same time, an anatomic organ and a spiritual symbol. It was characterized by two terms, *haty* and *ib*, and three fundamental concepts. 1) *Haty*, representing cardiac muscle, even if there was no a precise conception of “muscle” [17]. 2) *Ib* or internal-*ib*, corresponding to the content of thoracic and abdominal cavities, except the heart-*haty*. In some texts, we find that in the internal-*ib* of any individual there is a divinity or even that the internal-*ib* is a divinity itself [17]. The communication between *haty* and *ib* was guaranteed by a network of vessels, named *metu* (*met* at singular), which contained all bodily fluids. The heart-*ib* received sensorial information by sense organs and therefore it was the seat of emotions and intelligence [18]. 3) Finally, there was the spiritual heart, which represented the centre of character, thinking and memory.

All the feelings, conditions of the soul, and traits of character were expressed in Egyptian by various idioms referring to the heart, because the compound words included *ib* [21], [19], [20]. In Demotic, late stage of Egyptian language, also the term *haty* is connected to many expressions related to emotional states, thinking and personal character [22].

**Fig. 2:**
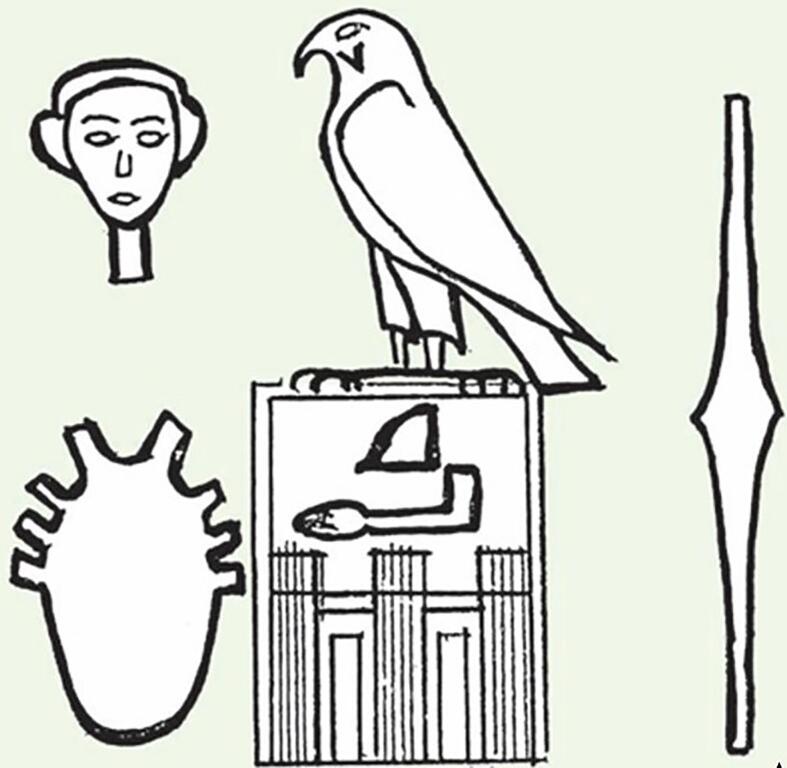
Ancient hieroglyphic representing the heart. Titulary of the Pharaoh Horus Qa (about 3000 BCE) where a precise anatomic illustration of the heart is represented, as a vase with eight handles, the disposition of which recall the anatomic position of aorta, pulmonary artery, superior and inferior vena cava and four pulmonary veins.

The most ancient hieroglyphic of the heart is found in the titulary of the Pharaoh *Horus Qâ* dating back to the first dynasty (about 3000 BCE). It corresponds to an anatomic illustration extremely precise which has no equal in any other coeval civilization (Fig. 2) [18], [23]. It represents a vase with eight handles, which recalls the anatomic position of aorta, pulmonary artery, superior and inferior vena cava and four pulmonary veins.

The cardiovascular physiology was based on three elements: *metu*; heart-*haty*; and the interior-*ib*. *Metu* could indicate not only blood vessels, but also tendons and muscles, particularly those long and thin. Because of muscle contractility, *metu* were also compared to cords [24]. They might also include nerves, but it is unclear whether ancient Egyptians had any concept of the nervous system [19]. *Metu* was an original Egyptian concept and it referred to any “conduit” of the body charged with one of more liquids [17].

In the “Vessel book” or “Book of Heart”, it is written that the heart “speaks” to all the parts of the body through the *metu* (paragraphs 854 and 856 of Ebers papyrus, and paragraph 163 of Berlin papyrus). The “*knowledge of the heart’s movement*” mentioned in that manuscript, means literally that the heart could move within the body and any change from its proper position was correlated to a pathological condition [17]. The heart’s ability to speak was related to the air that accompanied blood and other fluids in the “vascular system” [16]. In the Smith papyrus, a sort of measurement of the “pulse” is mentioned [16]. In the Ebers, the relationship between the heart beat and the pulse is confirmed: “*The interior-ib is weak if the heart-haty does not speak anymore or if the metu of the heart-haty are silent and they don’t give any indication to the hands*” [18]. This seems the description of a cardiogenic shock.

The air inspired thought the trachea was the element by which the heart spoke with vessels [19]. Therefore, air was responsible of the contraction of the heart and vessels [16]. Through the *metu*, also the “breath of life” and the “breath of death” were transported [19].

The strict relationship between heart and *metu* favoured the development of the tactile arterial palpation of the heartbeat by fingertips. Egyptian doctors believed that *metu* had to be soft and elastic for fulfilling their function and that a state of rigidity might represent a pathological condition. To “measure” radial pulse, it seems that Egyptians used a grain measure specifically calibrated, as can be found in the Smith papyrus [13], [16].

As for pathological conditions, the heart could be weak because of *wekhedu* (accumulation of by-products of un-expelled bodily waste); heat; excess of blood; something entering from outside; anger; and wrong anatomical positions [19]. Some descriptions of “weakness” of the heart might refer to heart failure, related to a state of “flooding” of the heart with blood [19]. A quite recurrent pathological concept, connecting the heart-*haty*, the *metu* and the interior-*ib*, was related to an excess of air in the heart-*haty*, which, in turn, determined that “physiological” liquids clogged up the *metu* causing an overflowing affecting the organs of the interior-*ib* [17].

In the Ebers papyrus we find a famous description interpreted as referring to a case of angina pectoris [15], [20]. In this same papyrus, a trouble of consciousness was correlated to a heart disease [18]. Interestingly, in case of “*fever or inflammation of the heart*”, the author of the papyrus advances a reasonable advice: “*The heart during such disease must be made to rest to some extent if it be possible*” [25].

Egyptians used a great amount of natural remedies, from plants to minerals, for curing diseases. The use of honey, willow, and sodium is attested in Smith and Ebers papyri, but many other substances were employed, among them also opium poppy as painkiller [26]. As soporific, Egyptians used also the mandrake, containing the narcotics atropine and scopolamine, mixed with beer or wine [27]. Regarding the heart, dates and castor-oil plant were used for “refreshing” heart and vessels [28].

## 3 The heart in Vedic medicine and Ayurveda

In Vedic medicine (northern Indian subcontinent, ca 1500–600 BCE), the function of breathing seems to be connected to the health of the heart, preluding yoga techniques which would have developed in India centuries later [29]. The heart was compared to a lotus from which departed “9 doors”, which could correspond to the great vessels of the heart.

**Fig. 3:**
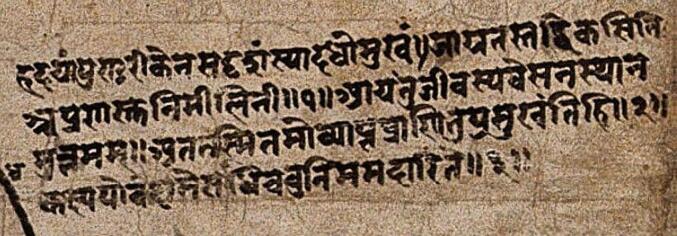
Caption of the “Nepalese anatomical figure” (ca. 1800) where the heart is described as follow: “The heart is similar to a spilled lotus. It opens upon awakening and it closes during sleep. Hence, when it is pervaded by torpidity, living beings fallen asleep”.

The heart was indicated with the Sanskrit term *Hridaya*, composed of three roots. *Hr-Harana*, “to receive”; *Da-Dana*, “to give away”; and *Ayana*, indicating the movement and the continuous maintaining of the previous functions [30], [31]. In the *Susruta* (known as *Susrutasamhita*, the oldest Indian work on surgery), the heart was defined as a “bud of flower lotus” with the apex facing down (Fig. 3). From an anatomical point of view, the heart was between the lungs. On its bottom left there was the spleen, and on bottom right the liver [32].

The heart was the origin of blood vessels, differentiated as *dhamanis*, *siras* and *srotas*. *Dhamanis* are “vessels that pulsate”; *siras* “vessels that carry the content without pulsating”; and *srotas* “vessels from which fluids move out” [33]. Through these vessels all fluids flowed, such as blood, lymph, faeces, semen, menstrual blood and water [34]. The fact that *dhamanis* originated from the heart and *siras* terminated in the heart, brought some scholars to advance that the idea of arterial (*dhamanis*) and venous (*siras*) blood flow was understood [30]. Similarly, some advance that the concept of systemic circulation was present in Ayurveda classic texts. In the *Caraka* (the book marking the dawn of Ayurveda, being a later version of the so-called *Agnivesh Tantra*, revised and improved by Caraka in a period between the 1st cent. BCE and 150 CE), for instance, the process of tissue nourishment was compared with a rotating wheel [33]. However, in classic Ayurveda texts, a clear description of circulation cannot be found.

*Caraka* advanced that for maintaining the health the cardiovascular system, it was necessary to avoid what we would call now as “stress” [35]. Among the therapeutic instruments for alleviating the “mental worries”, there was probably some form of meditation [35]. The doctor should advice his patients to breathing deeply and slowly, because other kind of breaths, more gasping or laborious, were signs of disease.

Ayurveda physiology was based on three principles, *Vata*, *Pitta* and *Kapha*, called *Dosha* (including several *sub-dosha*) partly resembling Western humoral conception. In the heart, three subtypes of Vata were located: Prana, Vyana and Samana. Then, a subtype of Pitta, the Sadhaka. Finally, a subtype of Kapha, the Avalambaka [32]. The concept of Prana is particularly important, being the vital blow acquired through respiration. Located in the heart, it controlled its movements and vitality. The Prana Vata is also situated in the head and it controls intellectual and cardiovascular functions, sense organs, psychological activities, respiration, and reflex activities. The heart was considered the centre of distribution of fundamental bodily fluids (blood, plasma and digested food) identified in the concept of Rasa. The impulse to the distribution was controlled by the Vyana. The Vyana Vata makes Rasa to get forcefully ejected out of the heart and makes it “circulate” throughout the body. Sympathetic and parasympathetic control of heart should be included under this subdosha [32]. Moreover, the Samana, located in the intestine, had the function of sending the Rasa rich of digested food (called Rakta) toward the heart, probably indicating the venous return of blood to the heart. Though there is no definitive information regarding heart’s cavities, it is clearly mentioned that the heart is a hollow organ giving space to Rasa and Rakta, which are constantly flowing inward and outward [30]. Again, in the brain and in the heart an important subtype of Pitta, that is, the Sadhaka, was located. It was responsible of the principal functions nowadays correlated to brain, that is, intelligence and memory [30], [31]. It also acts on heart as a cardiac stimulant [32]. In particular, Sadhaka controlled Medha, which indicated the intelligence; and Buddhi, which corresponded to the analytic skill of the mind. Finally, the Avalambaka, a subtype of Kapha, nourishes the aqueous part of the heart through the energy taken from food, giving it force and resistance.

With regard to heart diseases, called *Hridaya Roga*, they were connected to disorders in diet, mental worries, and to excessive of exercise or, conversely, sedentary habits. This vision seems not far from what contemporary Western medicine has established as risk factors connected to cardiovascular diseases. These features can cause different cardiovascular symptoms that can be summarized in three main categories: pain, dyspnoea and oedema [30].

In classic Ayurveda texts, about 200 drugs for cardiovascular diseases are mentioned, and among them the most frequent ingredients are Piper longum, Piper nigrum and Zingiber officinale [36]. Ayurveda still nowadays employs a series of medicinal herbs which seems to have a certain therapeutic potential. For instance, several studies has been carried out on the hypocholesterolemic, hypolipidemic, anti-thrombotic and antiatherosclerotic properties of the traditional Ayurvedic herbs Commiphora mukul (Guggulu), Allium Sativum (Rasona) and T. arjuna (Arjuna) with encouraging results [30]. Interestingly, the Guggulu seems to produce also electrocardiographic improvements through its action on the thyroid gland [30].

## 4 The heart in traditional Chinese medicine

Traditional Chinese Medicine developed from around 2700–2200 BCE to the beginning of the Imperial Unity (dynasty Qin 221–206 BCE). In the *The Yellow Emperor’s Inner Canon of Medicine* (attributed to the legendary emperor *Huang Di*, but written in a period between the end of the 2nd century BCE and the 2nd century CE), is stated that the heart, situated in the thorax, had a beat that can be seen or felt under the left nipple, serving as the source of the pulse beat [37]. The heart governed the blood and the lungs had a special function in absorbing the *qi*, the vital energy of the body and the cosmos, a sort of psycho-physical matter characterized by different degree of thickening.

The *qi*, in turn, had also the function of pushing the blood all around the body. Indeed, the relationship between heart and lungs was considered fundamental for the functioning of the cardiovascular system [38].

**Fig. 4:**
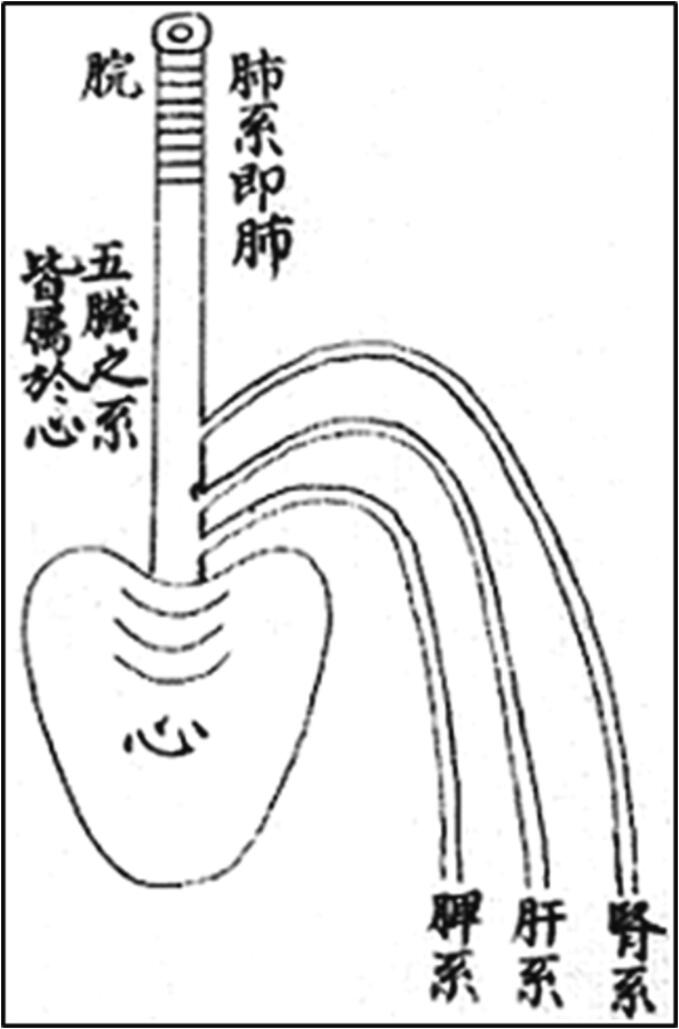
The heart in an 16th cent. Illustration of the Huangdi. The three lines indicates the connections between kidneys, liver and spleen. The central conduit penetrating into the heart establishes the connection with lungs.

From the heart, three principal conduits originated, connected with kidneys, liver and spleen (Fig. 4). Given that the element of the heart was fire and the element of kidneys was water, the warmness of the heart served to equilibrate the coldness of kidneys, and vice versa, otherwise there could be a pathological imbalance causing several pathologies, like insomnia, anxiety disorder, menopausal syndrome, etc. [39].

The vascular system principally consisted in twelve pairs of vessels carrying blood, *qi* and air. Transliterated *mo*, or *mai*, they are variously translated as “channels”, “vessels”, or “pulse” [40]. Vessels were named *chin*,*lo*, and *sun*, interpreted as arteries, veins, and capillaries, but this is a posteriori interpretation, because no proper distinction was made between arteries and veins [41].

Heart is often described as “imperator”, “monarch”, “governor” [42]. It was the principal seat of the *qi*, the blood, and the *shen*, that is, the spirit or the human mind. Controlling blood, heart governed also body fluids and, with the lungs, transpiration. Armpits and palms were the points where perspiration, correlated also with emotionality, was more abundant, because these parts were crossed by the “heart canal”.

*Qi*, blood (*xue*) and *shen* were considered, with the *jing* (essence) and *jin yè* (bodily fluids), as the five “vital” substances of the human body [43]. They were manifestation of the *qi* according to different degrees of “thickening”: from the *jin yè*, the most “material”, to the *shen*, purely spiritual [38]. *Qi*,*shen* and *jing* are called also the “three treasures”, where *qi* and *shen* were considered as responsible of the psychic constitution, while the *jing* was correlated with the physical make-up of the body [44]. *Shen* was responsible of affectivity, thinking, conscience, memory and sleep. If the heart was weak, mental problems could emerge, such as depression, weak memory, sleepiness, insomnia, and loss of consciousness. All emotions affect the heart and the mechanism of the *qi*. The emotional stress, for instance, causes a stasis of *qi* that, in turn, determines an excess of heat and fire that, in the long term, dries out the *shen*, causing anxiety and agitation [40]. An excessive joy, understood as a state of excessive agitation and concupiscence, can move the *shen* from its natural seat, causing a dilatation of the heart [40].

The relationship between heart, *qi*, blood and blood vessels is at the base of the complex Chinese theory of “pulses” [45]. If the *qi* of the heart is strong, blood vessels are in good condition and the pulse is perceived as full and regular. The state of repletion or depletion of blood vessels, could signal illness, and had to be examined at locations in various parts of the body, particularly in the radial pulse. Its study was subdivided in three sectors and five levels of deepness [46]. All positions were in relation with the “canals” of “living *qi*” and were fundamental for acupuncture [47].

Any disturbance (excess, stasis or defect) of *qi*, blood and blood vessels, emotions and *shen*, and their circulation in the body could cause a heart disease. Emotions, for instance, were considered the principal “internal” causes of heart diseases. Sadness could exhaust the *qi* of both heart and lungs, a condition which can be detected with a slow pulse [38].

Finally, among the great number of studies realized in recent years for testing traditional Chinese herbal medicines [48], there is the compound *Xuanbi antong* granule for treating borderline coronary lesion (BCL). In general, for coronary heart disease, traditional Chinese herbal medicine uses *Salvia miltiorrhiza*, and current studies show that it could relieve small artery circulation, restrain cell apoptosis, and protect the heart against ischemia–reperfusion injury.

## 5 *Relaxation response* through breathing control and avoiding mind wondering

Due to lack of space, in that paper we did not mention treatment for cardiovascular diseases which included a ritual part based on the recite of incantations and magic formula, often accompanied by drums or music. In a Sumerian incantation against the demon *udug*, correlated to a heart constriction, the physician was requested to “calm the patient” by using a copper drum, called “hero of heaven”, the noise of which “drives away evil”. A hymn of the Atharvaveda (one of the main texts of Vedic medicine) celebrated the ritual charm as the principal instrument against diseases. Another hymn was dedicated to blood vessels, invoking their state of calm. The Vedic medical men *bhisaj*, because of dancing and music, was also called shaker and chanter.

First, these formulas could have been useful in eliciting the placebo effect. By containing statements about their force and effectiveness, they favoured the conviction in the patient to be in the best setting for curing illness. Since the 1970 s, an important physiological reaction has been discovered that can shed further light on the biological efficacy of ancient magical-therapeutic treatments. That the human body, in case of perceived danger, activate the so-called *stress response*, was well known, as well as that a chronic activation of this survival mechanism impairs health. The American cardiologist Herbert Benson (1935–2022) demonstrated that the human body disposes of a complementary reaction: the *relaxation response* [49]. It is based on a state of relaxation of mind, which can be activated by slow and deep breathing, and by the repetition of a simple word or phrase. This last activity keeps the mind from “wandering”, reducing psychic stress and activating the parasympathetic nervous system, which, in turn, slows heart rate, lowers blood pressure, and improve immune system. The combined control of thought and breathing forms the basis of meditation techniques, as elaborated, since the 5th century CE, by the Yoga, which is the most renewed among these forms of mental concentration. Benson, in fact, founded a *Mind-Body Medical Institute*, renamed *Benson-Henry Institute*, at Massachusetts General Hospital in Boston, where meditation is regarded as synonymous of relaxation response. We can affirm that even the magical-therapeutic practices of ancient medicines favoured the activation of the relaxation response. Magico-therapeutic rituals were based on repetitive formulas which often have to be recited by both shamans-doctors and patients. In these latter, the recitation might have favoured the concentration of mind, keeping from wandering, as well as a deep and slow breathing.

In other terms, it is true that the cardiovascular system was surely not well understood anywhere in antiquity. The heart was not understood as a pump, but remained the center of mind and emotions, since the brain as an organ was also largely misunderstood. However, ancient physicians were aware that we “feel” emotions in our chest rather than in our brains, and that feeling is strictly connected with breathing. Indeed, they developed the idea of a strict connection between mind, emotion, breathing, and heart, which today is at a center of interesting perspectives in neuro-cardiovascular research.

## CRediT authorship contribution statement

**Fabio Zampieri:** Conceptualization, Investigation, Writing – original draft. **Gaetano Thiene:** Writing – review & editing. **Alberto Zanatta:** Validation, Visualization, Writing – review & editing.

## Declaration of Competing Interest

The authors declare that they have no known competing financial interests or personal relationships that could have appeared to influence the work reported in this paper.
